# OLED Structure Optimization for Pure and Efficient NIR Electroluminescence of Nd^3+^ Complexes Bearing Fluorinated 1,3-Diketones

**DOI:** 10.3390/ma16031243

**Published:** 2023-02-01

**Authors:** Daria A. Metlina, Dmitry O. Goryachii, Mikhail T. Metlin, Lyudmila V. Mikhalchenko, Vladislav M. Korshunov, Ilya V. Taydakov

**Affiliations:** 1 P.N. Lebedev Physical Institute of the Russian Academy of Sciences, 53 Leninsky Prospect, 119991 Moscow, Russia; 2 N.D. Zelinsky Institute of Organic Chemistry of the Russian Academy of Sciences, 47 Leninsky Prospect, 119991 Moscow, Russia; 3 G.V. Plekhanov Russian University of Economics, 36 Stremyanny per., 117997 Moscow, Russia

**Keywords:** 1,3-diketone, neodymium, NIR OLED, coordination compounds, electroluminescence

## Abstract

NIR emitting OLEDs (organic light-emitting diodes) with high photoluminescence quantum yields were developed on the basis of fluorinated 1,3-diketonate coordination compounds of the Nd^3+^ ion. Both thermal evaporation and spin-coating techniques were successfully employed for active layer deposition resulting in electroluminescence quantum yields up to 1.38·10^−2^%. Blueish-green emission from exciplex and electroplax formations was almost suppressed with the topology optimization of the cell.

## 1. Introduction

To date, NIR (near-infrared) emitting lanthanide coordination compounds, specifically those with Nd^3+^, Er^3+^, and Yb^3+^ ions, are extensively employed in advanced photonics, optoelectronics, and spectroscopy [1,2,3,4,5,6,7]. This special interest is explained by the narrow luminescent bands observed in the 880–1600 nm spectral range originating from these ions. Particularly, the Nd^3+^ emission bands at 880, 1060, and 1330 nm fall within the telecommunications range and the transparency region of biological tissues [8,9,10]. Moreover, the electroluminescent properties of lanthanide complexes are beneficial for OLED design, since 75% of emission in these devices is produced via recombination of electrons and holes that forms triplet excitons [11]. Triplet harvesting is possible in high-cost iridium complexes [12,13] and TADF (thermally activated delayed fluorescence) materials [14,15]; however, their luminescence bandwidth is broader than 100 nm, while iridium complexes have emissions only at wavelengths shorter than 800 nm [7,16]. Alternatively, this effect is possible due to the “antenna-effect” [17] in much cheaper Ln^3+^ compounds with π-conjugated ligands such as 1,3-diketones with sharp luminescent bands.

Many efforts were made in the efficient design of NIR-OLEDs based on 1,3-diketone neodymium complexes; however, this is still a challenging task, and after the electroluminescence of a Nd^3+^ complex was demonstrated in 1999 [18], only a few articles with highly efficient devices were reported [19,20,21]. First, the efficiency of OLED emission depends on the photoluminescence quantum yield (PLQY) of the emitter; in the case of lanthanide compounds, PLQY strongly depends on the difference between the triplet energy level of the ligand environment and the resonant acceptor level of the Ln^3+^ ion [17]. The corresponding energy gap is usually wide (>>10,000 cm^−1^) in the case of Nd^3+^ and other NIR-emitting Ln^3+^, and nonradiative energy relaxation should be minimized by a proper choice of ligands. Second, the luminescence of all lanthanide complexes is sensitive to the multiphonon relaxation caused by the presence of CH and OH groups in the ion coordination sphere [22,23]. Replacement of these high-frequency-oscillating groups with low-frequency-oscillating ones, e.g., deuterated or fluoro-containing groups [24], makes it possible to suppress luminescence quenching. Successive elongation of fluorinated chains in the ligands can additionally increase the efficiency of lanthanide-centered luminescence [23]. In this respect, the 1,3-diketonate ligands proved to be convenient for highly luminescent lanthanide complexes [25]. Finally, coordination compounds should have high volatility and/or solubility in various solvents to be suitable for OLED design, and 1,3-diketones usually satisfy these conditions [26,27].

**Figure 1:**
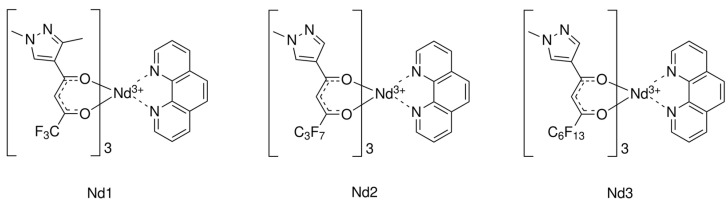
The 1,3-diketonate coordination compounds of Nd^3+^ synthesized.

The successful design of efficient multilayer OLEDs also requires an accurate selection of layer materials and a proper choice of the emission layer deposition technique (spin-coating or thermal evaporation). Thermally evaporated layers commonly allow higher electroluminescence efficiency to be achieved, but the spin-coating method is cheaper and more suitable for mass production [28,29,30], thus most of the OLEDs with neodymium coordination compounds were designed using the latter technique [19,20,21]. In a study of the photoluminescence of novel neodymium coordination compounds with a new type of 1,3-diketone containing linear C_x_F_2x+1_ groups (x = 1,3,6) and a pyrazole moiety, as well as a 1,10-phenanthroline ancillary ligand, see Figure 1, we observed a strong Nd^3+^-associated NIR luminescence with PLQY up to 1.08% [31], close to the highest values reported for other Nd^3+^ complexes with 1,3-diketonate ligands. Moreover, their electroluminescent application in OLEDs was successfully tested in a thermally deposited structure based on the **Nd2** complex with x = 3. In a recent study aimed at improving our results, we undertook comprehensive efforts for a series of complexes by modifying the OLED structure with various electron/hole transport materials and compared the thermal evaporation and spin-coating methods for emissive layer deposition.

## 2. Materials and Methods

A recent study was performed for a series of pyrazole substituted 1,3-diketonate neodymium(III) coordination compounds, tris(1-(1,3-dimethyl-1H-pyrazol-4-yl)-4,4,4-trifluorobutane-1,3-dionato)(1,10-phenanthroline) neodymium(III) (**Nd1** complex), tris(1-(1-methy-1H-pyrazol-4-yl)-4,4,5,5,6,6,6-heptafluorohexane-1,3-dionato)(1,10-phenanthroline) neodymium(III) (**Nd2** complex), and tris(4,4,5,5,6,6,7,7,8,8,9,9,9-tridecafluoro-1-(1-methyl-1Hpyrazol-4-yl)nonane-1,3-dionato)(1,10-phenanthroline) neodymium(III) (**Nd3** complex), shown in Figure 1. Synthetic details can be found in the Supplementary Materials.

The main ligands, 1-(1,3-dimethyl-1H-pyrazol-4-yl)-4,4,4-trifluorobutane-1,3-dione (**HL_1_**), 1-(1-methyl-1H-pyrazol4-yl)-4,4,5,5,6,6,6-heptafluorohexane-1,3-dione (**HL_2_**), and 4,4,5,5,6,6,7,7,8,8,9,9,9-tridecafluoro-1-(1-methyl-1H-pyrazol-4-yl)nonane-1,3-dione (**HL_3_**), were synthesized by the previously reported method [32]. All the other reagents were purchased from Aldrich and used without further purification. Elemental analysis was performed on an Elementar CHNO(S) analyzer. Fourier-transform infrared spectroscopy (FTIR) spectra were obtained on a Bruker Vector 22 instrument in KBr pellets. Neodymium was determined by complexometric titration with a standard Trilon B solution in the presence of Xylenol Orange as the indicator. The complexes were decomposed by heating with 70% HNO_3_ before the analysis.

Quantum-chemical calculations were performed with the Gaussian 16 Rev A.03 program [33]. Density-functional theory (DFT), time-dependent DFT (TD-DFT) and Tamm–Dancoff approximation (TDA) calculations at B3PW91/6-311+G(d,p) level of theory [34,35] were employed for all the atoms except lanthanides. Large-core energy-adjusted relativistic energy-consistent pseudopotential (RECP) for Nd, developed by the Stuttgart and Dresden groups, along with the accompanying basis set ECP49MWB28 was used. In the development of the theoretical model, the geometrical parameters of X-ray single crystal structures were used as the starting point. All the calculations were performed in the gas phase. Analysis of vibrational frequencies was performed for all the optimized structures. All the compounds were characterized by real vibrational frequencies only. The wavefunction stability using a stable keyword was also checked for ground state geometry.

Thermogravimetry differential thermal analyses (TG-DTA) were performed on a NETZSCH STA 409 PC/PG instrument using an Al_2_O_3_ crucible, with sample masses of approximately 4.3, 3.9, and 4.1 mg for complexes **Nd1**, **Nd2**, and **Nd3**, respectively, at a heating rate of 10 C·min^−1^ and in dry air atmosphere with a flow rate of 30 mL·min^−1^, in the temperature range between 40 and 750 °C. 

Cyclic voltammograms (CVs) were recorded with an IPC-Pro potentiostat (Econix, Russia) using a 0.1 M solution of tetrabutylammonium tetrafluoroborate in CH_3_CN as the supporting electrolyte. The measurements were performed with a glassy carbon working electrode and a platinum grid auxiliary electrode, and a saturated calomel reference electrode connected to the solution in the cell via a ceramic membrane bridge filled with the supporting electrolyte. The peak potentials were determined relative to the redox potential of Fc/Fc^+^. Ferrocene as the internal standard was added to the solution after recording the curves of the compounds studied. The solution was purged with high-purity argon to remove dissolved oxygen. The curves were recorded at a potential sweep rate of 0.1 Vs^−1^. 

Near-IR photoluminescence and excitation spectra of the complexes were measured at room temperature on a Horiba JobinYvon Fluorolog QM spectrofluorimeter using a 75 W xenon arc lamp. The experiments were carried out in DMSO solutions of the complexes poured into quartz cells.

The OLED structures were developed with poly(3,4-ethylenedioxythiophene):poly(styrenesulfonic acid) (PEDOT:PSS) hole injection material (Lumtec LT-PS001), Tris(4-carbazoyl-9-ylphenyl)amine (TCTA) host material, and 2,2′,2”-(1,3,5-Benzinetriyl)-tris(1-phenyl-1-H-benzimidazole) (TPBi) (Lumtec LT-E302) electron transport material, as well as LiF (Lumtec LT-E001) and Al cathode materials. All of them were used without further purification. ITO-coated glass (Lumtec, Taiwan) substrates with 12 Ohm/sq resistance were successively cleaned by ultrasonication in 15% KOH alcoholic solution, double distilled water, and isopropanol for 10 min each, followed by drying with a dust-free nitrogen flow. The substrates were additionally treated by UV/ozone in a UV-cleaning chamber (Ossila, UK) for 25 min just before use. 

Deposition of layers was performed as follows. First, 200 µL of an aqueous solution of PEDOT-PSS film was spin-coated at 2000 rpm for 1 min onto a freshly prepared ITO substrate, and the resulting anode was annealed at 130 °C for 20 min in Ar atmosphere (in a glovebox) to form a 40 nm PEDOT-PSS film. 

**Figure 2:**
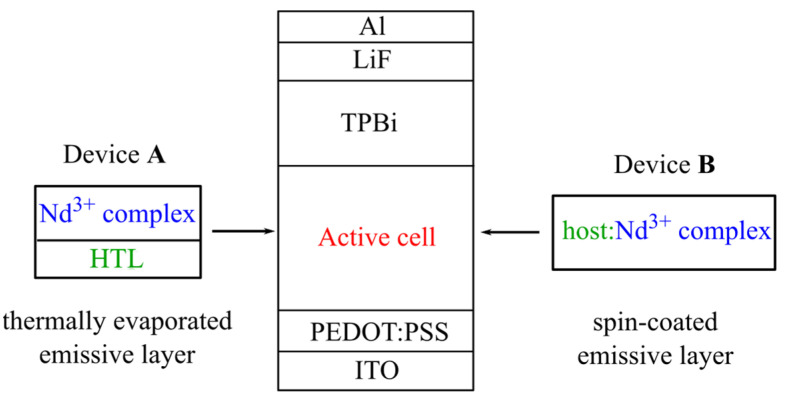
Topology of the OLED devices designed with the **Nd1**, **Nd2**, and **Nd3** complexes as emitters.

A series of **A** type OLEDs, see Figure 2, were obtained with hole transport layers (HTL) of PVK (Sigma-Aldrich, a hole transport and hole injection layer), CBP:TCTA (7:3) or poly-TPD (Ossila, UK) spin-coated from a chlorobenzene solution (5 g/L) directly onto the PEDOT:PSS-coated substrate at 2000 rpm followed by drying at 130 °C for 8 min. Emissive layers of pure complexes (20 nm) were thermally evaporated from a quartz crucible heated with a tantalum coil at a deposition rate of 0.1/s. A TPBi was thermally deposited in a vacuum of 10^−3^ Pa from a quartz crucible heated with a tantalum coil. The layer thickness was 15 nm. Finally, a composite cathode consisting of LiF (1 nm) and aluminum (40 nm) was thermally deposited through a shadow mask to form four active pixels of 12 mm^2^ at the rates of 0.1 and 0.2 nm/s, respectively.

OLEDs of **B** type were designed with the [host material]:[Nd^3+^ complex] emission layer spin-coated from a chloroform solution onto PEDOT-PSS at 2000 rpm and dried at 100 °C for 20 min. PVK, TCTA, and TCTA:CBP mixture with a complex in 1:1 and 1:5 ratios were used as a host. Further deposition of an TPBi, LIF, and Al was performed in the same way as for the A devices.

Electroluminescence spectra were measured at room temperature with an Ocean Optics Maya 2000 Pro CCD spectrometer. The current–voltage characteristics were obtained using two DT 838 Digital multimeters. The deposition rate was measured in situ by a Leybold Inficon IC-6000 deposition controller calibrated using an NT-MDT atomic force microscope of Integra family. The emission power was measured with a FieldMaxII-TO power meter equipped with a high sensitivity optical power sensor 1,098,313 VIS (Coherent) operating in the 400–1600 nm range. 

In all the optical measurements, the corresponding instrument response functions were taken into consideration. The experiments were performed in air under atmospheric pressure. No degradation of optical properties was observed during the experiments. 

## 3. Results and Discussion

### 3.1. Photoluminescence Properties

**Figure 3:**
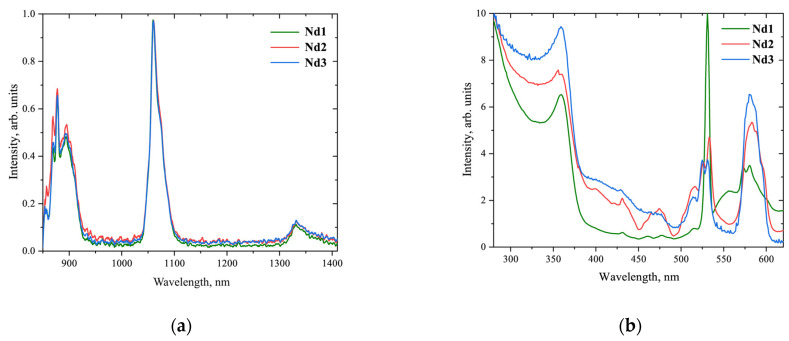
Photoluminescence spectra (365~nm excitation wavelength) (**a**) and excitation spectra (recorded at 1060~nm) (**b**) for complexes **Nd1**-**Nd3** in DMSO solutions with a concentration of 3·10^−3^ M.

**Figure 4:**
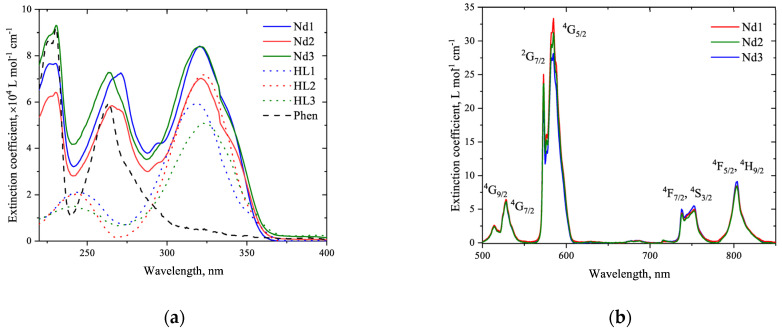
Absorption spectra in UV region (**a**) and Vis–NIR region (**b**) for acetonitrile solutions of **Nd1**-**Nd3** complexes, 1,10-phenanthroline ligand (phen), and pyrazolic 1,3-diketonate ligands with C_x_F_2x+1_ groups (**HL_1_**-**HL_3_**; x = 1, 3, 6). The concentrations were 5 ·10^−5^ M and 5·10^−3^ M.

The synthesized **Nd1**-**Nd3** neodymium complexes were proved to be intense NIR-emitters under UV excitation at 365 nm; see Figure 3a [31]. Luminescent bands characteristic of ^4^F_3/2_ → ^4^I_9/2_ (880 nm), ^4^F_3/2_ → ^4^I_11/2_ (1060 nm), and ^4^F_3/2_ → ^4^I_13/2_ (1334 nm) transitions in neodymium ion are observed with a maximum intensity at 1060 nm. Due to the low extinction of the Nd^3+^ ion (below 40 mol^−1^·cm^−1^), this photoluminescence resulted from the “antenna effect” with excitation of the complexes via absorption bands of the ligand environment in the UV–vis region; see Figure 4a and Figure 3b. Bonding pyrazolic 1,3-diketonate and 1,10-phenanthroline ligands to Nd^3+^ gives complexes with intense absorption, their extinction coefficients reaching 10^5^ mol^−1^·cm^−1^; see Figure 4b. As a result, the measured PLQYs of our complexes in solutions were 0.93 ± 0.09%, 0.75 ± 0.08% and 1.08 ± 0.11% for **Nd1**, **Nd2,** and **Nd3**, respectively [31]. These values exceed the commonly reported values near 0.6% for other 1,3-diketonate neodymium complexes [36,37,38], which encouraged us to study the electroluminescent properties of **Nd1**-**Nd3**. The PLQYs for our complexes in thin films were measured to be slightly lower than those in solutions—0.81 ± 0.11%, 0.58 ± 0.09%, and 0.92 ± 0.09% for **Nd1**, **Nd2**, and **Nd3**, respectively.

### 3.2. Thermogravimetric Analysis (TGA) and Differential Thermal Analysis (DTA)

**Figure 5:**
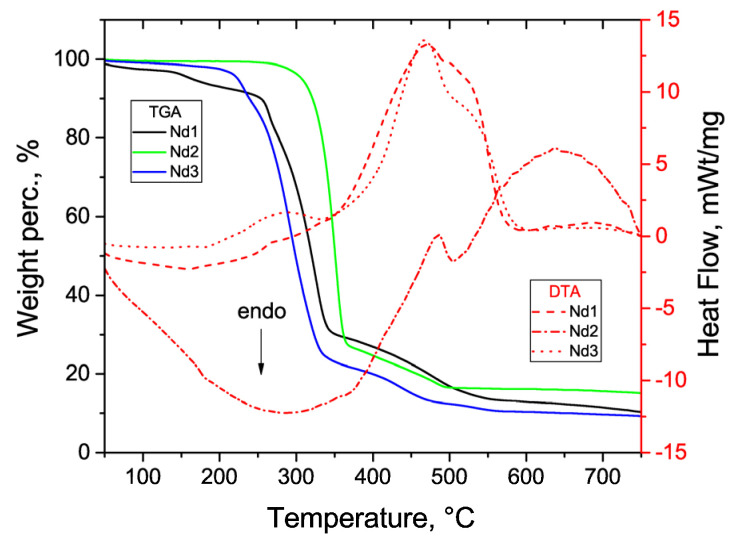
TGA and DTA curves of complexes **Nd1**, **Nd2** and **Nd3**.

The thermograms of all the neodymium complexes (see Figure 5) are almost similar in shape and show two gradual main steps of weight loss (WL), which are consistent with decomposition and combustion of the organic ligands. The complexes are stable up to 225 °C (**Nd1**), 250 °C (**Nd2**), and 190 °C (**Nd3**), which is beneficial for OLED designing. Further heating entails a melting stage with WLs of 67%, 75% and 77% for **Nd1**, **Nd2**, and **Nd3**, respectively, associated with the removal of the main ligands, see Table S1. Complexes heated to temperatures higher than 350 °C decompose exothermally; WLs of 17%, 12%, and 11% may indicate the removal of the 1,10-phenanthroline molecule with Nd_2_O_3_ formation above 500 °C (the calculated WLs are 17%, 14%, and 10% of an ancillary ligand for **Nd1**, **Nd**2 and **Nd3**, respectively). The thermal effects can be attributed as follows: endothermic processes at 200–350 °C are associated with melting, the first exothermic process at 425–600 °C is associated with burning, and the second exothermic process at 650°C with subsequent recrystallization of NdF_3_.

### 3.3. TD-DFT Analysis

The triplet energy level of the ligand is essential for complexes with efficient emission. The first excited triplet state energy (T1) value was estimated by TD-DFT calculations for the optimized geometry of the **Nd1** complex, only since the crystals suitable for X-ray structural analysis could not be grown from the **Nd2** and **Nd3** complexes [31]. 

The calculated energies of the lowest unoccupied (LUMO) and the highest occupied (HOMO) orbitals are E_HOMO_ = −6.13 eV and E_LUMO_ = −2.53 eV, while 3.87 eV is determined by the maximum of the highest-energy band in UV–vis absorption spectra of this complex energy gap. The lowest energy transition S_0_–T_1_ has the major contributions HOMO-2 to LUMO+2 and HOMO-3 to LUMO+4 located on three **L_1_** molecules in **Nd1** (see Figure S6, Table S2). Therefore, the first excited triplet state T_1_ of the complex is predominantly localized on these molecules. Notably, the CF_3_ radical does not participate in the occupied molecular orbitals and slightly participates in the unoccupied ones. Hence, the fluorinated carbon chain of the **L_1_** ligand has a slight contribution to the π–π∗ transition (S_0_–T_1_), and therefore has an impact on the T_1_ state of the **L_1_** ligand. T_1_ energies of 22271 and 23095 cm^−1^ were calculated for the **Nd1** complex using the TD and TDA methods, respectively; see Table 1. These values are in good agreement with the experimental value previously determined as 22055 cm^−1^ [31].

### 3.4. Electrochemical Properties

**Figure 6:**
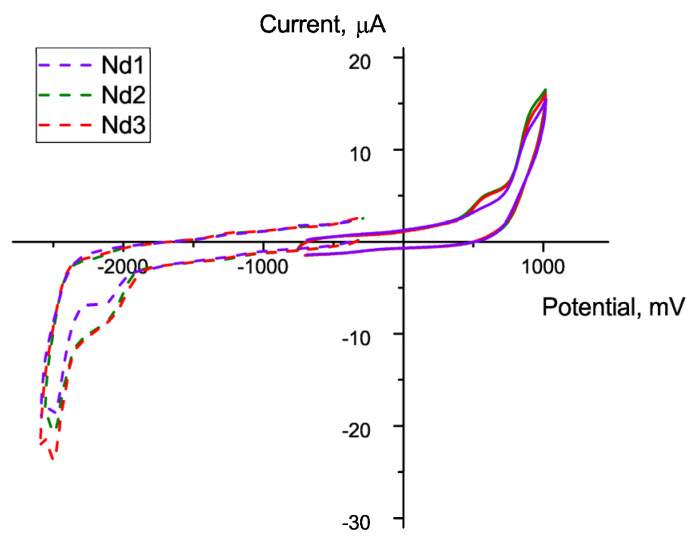
Electroreduction (dashed) and electrooxidation (solid) CV curves of the **Nd1**, **Nd2** and **Nd3** complexes measured on glassy carbon working electrode in DMF (0.1 M Bu_4_NBF_4_) solution at a potential sweep rate of 0.1 Vs^−1.^

The energies of the frontier orbitals were determined using the values of the electrooxidation and electroreduction potentials from the CVs of the **Nd1**, **Nd2**, and **Nd3** complexes. All the three compounds evince similar electrochemical properties. The first stages of electroreduction and electrooxidation of the complexes are irreversible at a potential scan rate of 0.1 Vs^−1^, see Figure 6. The energies of the LUMO and HOMO orbitals were calculated from the magnitudes of the electroreduction (E^red^) and electrooxidation (E^ox^) peaks onset, respectively. The E^red^_onset_ and E^ox^_onset_ values were estimated relative to the ferrocene/ferrocenium (Fc/Fc^+^) reversible oxidation potential with an absolute value of −5.1 eV. The HOMO and LUMO values were determined according to [39] (see Equations (1) and (2)).

E_HOMO_ (eV) = −|e|(E^ox^_onset_ + 5.1 eV), (1)

E_LUMO_ (eV) = −|e|(E^red^_onset_ + 5.1 eV) (2)

The results of electrochemical studies are presented in Table 2. 

### 3.5. Electroluminescence

#### 3.5.1. Devices with Thermally Evaporated Layers of Nd^3+^ Complexes (**A**)

The efficient NIR emission for OLEDs based on 1,3-diketonate lanthanide coordination compounds drastically depends on the transport layer materials. 

**Figure 7:**
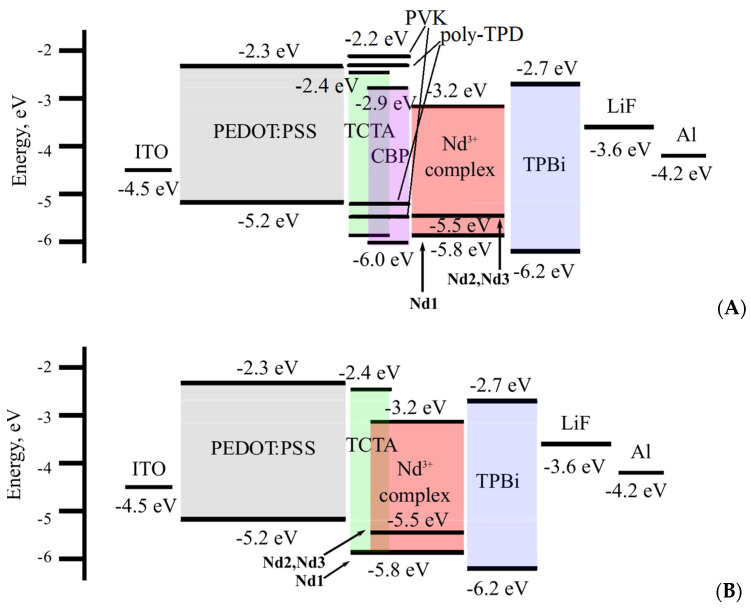
Energy level diagrams of the (**A**) and (**B**) devices with the **Nd1**, **Nd2** and **Nd3** complexes.

We developed **A** test devices based on the thermally evaporated **Nd1** complex using various hole-transport materials, namely, PVK, poly-TPD, and CBP:TCTA (7:3). The electroluminescent (EL) spectra of these diodes have NIR emission bands with maxima at 810, 890, and 1060 nm, characteristic of Nd^3+^ ion transitions (^4^F_5/2_+^2^H_9/2_→^4^I_9/2_, ^4^F_3/2_→^4^I_9/2_, ^4^F_3/2_→^4^I_11/2_, respectively; see Figure S1). Moreover, a broad band in the 400–800 nm range with maxima at 579, 610, and 623 nm is observed for OLEDs with PVK, CBP:TCTA and poly-TPD hole-transport layers (HTL); see Table 3. According to the electroluminescence spectra of the transport layers [40,41,42] and photoluminescence (PL) of the neat **HL_1_** ligand, this band should originate not from transitions in pure materials but from their electro-induced formations; the minor luminescent band observed at 415 nm is associated with the main ligand and/or transport layers; see Table 3. The maximum EQE along with the minimum contribution in the visible range of the spectra was obtained for the structure with CBP:TCTA, thus we chose this material to produce a series of OLEDs with similar structures for all three Nd^3+^ complexes; see Figure 7. 

**Figure 8:**
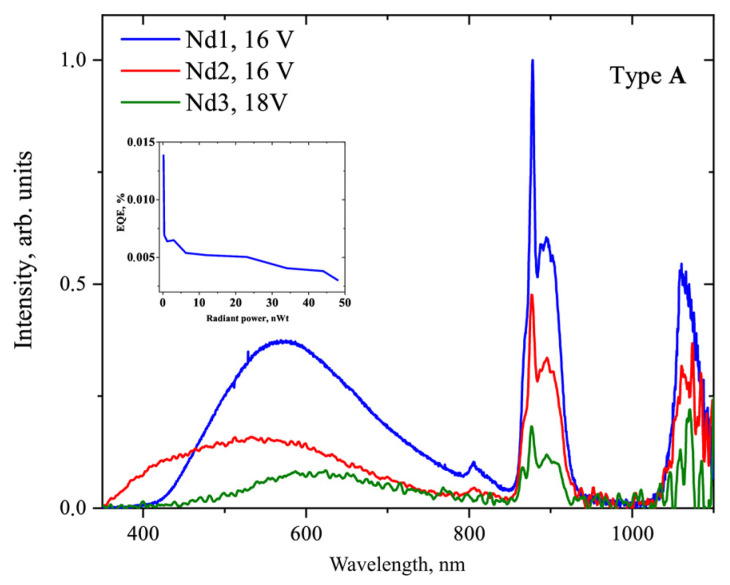
Electroluminescence spectra for A type devices based on the **Nd1**, **Nd2**, and **Nd3** complexes. The EQE–radiant power curve for OLED with the **Nd1** complex is shown in the inset.

The EL spectra of these diodes resemble each other (see Figure 8), with NIR luminescence originating from the Nd^3+^ ion. The intensity of the additional band in the visible range decreased in the series **Nd1**-**Nd3**-**Nd2** with a slight blue shift of the maximum from 610 nm to 550 nm. An EQE value of 1.38·10^−2^% was obtained only for the OLED based on the **Nd1** complex; see Figure S2. According to the TGA/DTA analysis, all the complexes are thermally stable to the point of 200°C. However, the other two devices were not suitable for EQE measurements. Apparently, the **Nd1** complex with the shortest fluorinated chain is more volatile at low temperatures than **Nd2** and **Nd3** with longer chains. It should be noted that upon thermal evaporation conditions only **Nd1** complex form high quality films, suitable for subsequent multilayer OLED fabrication. At the same time, thermally evaporated layers of **Nd2** and **Nd3** complexes were of substantially lower quality, which led to fast degradation of OLED structures under voltage. Thus, spin-coating appears to be more appropriate for the deposition of lanthanide complexes in terms of thermal stability [44].

#### 3.5.2. Devices with Spin-Coated Layers with Nd^3+^ Complexes (**B**)

Previously [31] we attempted to make OLEDs with the **Nd2** complex using the spin-coating technique for active layer deposition. It was shown, that wet fabricated OLEDs, based on complex **Nd2**, are much more stable and robust than ones, fabricated by thermal evaporation technique. 

**Figure 9:**
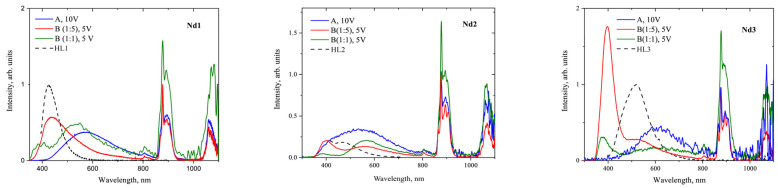
Comparison of the electroluminescence spectra for thermally evaporated **A** structures, and **B** structures with a spin-coated [complex:TCTA] layer with 1:1 and 1:5 ratios. The photoluminescence spectra of the neat main ligands are shown with dashed lines.

Continuing our previous work in order to achieve the maximum EQE value and the purest NIR EL spectra, we made **B** test structures with various materials of the spin-coated host matrix such as PVK, pure TCTA, and TCTA:CBP (3:7), and the **Nd2** complex as the guest. Compared to the **A** structures with the **Nd2** complex, the EL spectra of **B** have an additional blue EL with a maximum at ~420 nm—two times more intense than the luminescent band at 880 nm in the case of the PVK and TCTA:CBP hosts; see Figure S3. Although for all hosts the EL spectra of **B** devices with **Nd2** are similar in shape, employing TCTA instead of PVK or TCTA:CBP allows the blue emission to be suppressed considerably. At the optimal [host:guest] ratio of [5:1], the blue band intensity was almost zero for the diode with the TCTA host. So, this mixture was used to build **B** type OLEDs with a [TCTA:Nd complex] active layer. All three complexes **Nd1**-**Nd3** were tested in **B** type devices (see Figure 7B and Figure 9). It should be noted that purest NIR emission was observed for complexes **Nd1** and **Nd3** also at host:guest ratio of 5:1.

It should be noted that the broad band within 420–800 nm with a maximum at ~600 nm is observed in all the designed structures, both spin-coated and thermally evaporated. However, in the **A** series of OLEDs, the transport layers were spatially separated with the thermally evaporated neodymium complex. Thus, exciplex formation should occur at the interface between the transport layer and the active layer induced by the presence of the Nd^3+^ complex [45,46]. The reported electroluminescent spectra of OLEDs with an emission layer of CBP:([Eu(L_x_)phen], x = 1,2,3) and a TPBi electron transport layer [24] have only an Eu^3+^-associated narrow band with a maximum at ~615 nm. So, we performed additional experiments with the [Gd(L_2_)phen] complex to check whether the main ligands play a role in the exciplex formation.

The EL spectra of **B** diodes with a pure (TCTA) host and ([Gd(L_2_)phen]:TCTA) are similar in shape and have a blue EL band from 380 to 800 nm with a maximum at ~440 nm that may most likely be attributed to the TCTA-ETL exciplex formation. Replacing TPBi with BPhen material does not change the shape of the EL spectra with the peak position at ~460 nm; see Figure S5. In contrast, for the **B** OLED with the **Nd2** complex, replacement of TPBi for BPhen makes it possible to get rid of the blue electroluminescence band at ~440–460 nm. However, electric-field-induced formation between the transport layer and (**Nd2**:TCTA) was still observed as broad electroluminescence from 450 nm to 800 with a maximum at 590 nm, and this device was not suitable for EQE measurements.

The measured maximum EQE values for **B** OLEDs increase in the series **Nd1**–**Nd3**–**Nd2** up to 4.5 10^−3^% for the 5:1 host–guest ratio with the current density of 65 mA/cm^2^, of 67 mA/cm^2^, and of 60 mA/cm^2^; see Figure S4. The maximum power efficiency of the fabricated OLED structures was almost the same value of 0.1 μW/W, and the maximum obtained light power density corresponding to the integrated electroluminescence intensity of the infrared band was 0.1 μW/cm^2^ at 8 V. Notably, the PL quantum yields of the complexes increase in the same order [31]. Compared to the **A**-type OLEDs with the thermally evaporated **Nd1** complex and a turn-on voltage of 10 V, spin-coated series **B** OLEDs have about three times lower EQE, but the turn-on voltage is as small as 5–6 V. Factors such as complex orientation or aggregation could affect the operating voltage values, but most strongly the smaller ones resulting from the presence of a conductive matrix, since the surface roughness of spin-coated active layers was up to 2 nm; see Figure S7. The EQE values reported for other devices with spin-coated 1,3-diketonate neodymium coordination compounds as the active layer are of the same order (~10^−3^–10^−2^ [18,21,47]). Therefore, the synthesized complexes could be used as the active layer of operation-stable OLEDs, built by spin-coating deposition, and a proper choice of the cell materials makes it possible to obtain an almost pure NIR emission of the Nd^3+^ ion

## 4. Conclusions

A series of 1,3-diketonate neodymium complexes with a pyrazole moiety substituted with a fluorinated chain were successfully employed to design NIR-emitting OLEDs. Devices with thermally evaporated (**A**) and spin-coated (**B**) active layers both demonstrate NIR EL associated with the f–f transitions in the Nd^3+^ ion. The EQE value of 1.38·10^−2^%, with turn-on voltage of 10 V, obtained for A structures based on the **Nd1** complex with the CF_3_ substitutient, was close to the highest value reported in the literature. The commonly observed blue-green emission originating from electroplex formation between the transport layers and Nd^3+^ coordination compounds was almost suppressed by the optimization of the topology and host materials. It was shown that the **Nd2** and **Nd3** complexes with longer fluorinated chains were suitable only for spin-coated B structures. The EQE values of these devices with small turn-on voltages of 5–6 V increase in the same order as the PLQY of the pure complexes and reach 4.5·10^−3^% for **Nd2**.

## Figures and Tables

**Table 1:** The calculated energies of the first excited triplet state (T_1_) calculated for **Nd1** complex with the 6-311+G(d,p) basis set.

T1, Triplet State, nm/cm^−1^	Method	Major Contribution
449/22271	TD	HOMO-2- > LUMO+2 (42%) HOMO-3- > LUMO+4 (19%)
433/23095	TDA	HOMO-2- > LUMO+2 (44%) HOMO-3- > LUMO+4 (20%)

**Table 2:** Electrochemical properties of the investigated Nd^3+^ complexes in HCON(CH_3_)_2_ solution.

Sample	E^red^_onset_, V	E^ox^_onset_, V	E_LUMO_, eV	E_HOMO_, eV	Eg, eV
**Nd1**	−1.46	1.2	−3.15	−5.81	2.66
**Nd2**	−1.42	0.89	−3.18	−5.49	2.31
**Nd3**	−1.37	0.94	−3.23	−5.54	2.31

**Table 3:** Luminescent properties in visible spectral range for the materials employed in OLEDs.

Material	Luminescent Band, nm	Maximum, nm	Source
PVK	360–600	420	[40]
poly-TPD	400–550	430	[41]
CBP:TCTA	340–460	350; 380	[42]
TPBi	340–520	400	[43]
**HL1**	350–600	415	
**HL2**	350–700	410; 490	
**HL3**	400–750	520	

## Data Availability

Not applicable.

## Supplementary Materials

The following supporting information can be downloaded at https://www.mdpi.com/article/10.3390/ma16031243/s1: Synthesis of the complexes; Figure S1. Electroluminescence spectra for the **A** type devices based on the **Nd1** complex with **dmfl CBP:TCTA** (7:3, 2000 rpm) (1), **PVK** (2000 rpm) (2), and **polyTPD** (2000 rpm) (3). The photoluminescence spectrum of the neat **HL_1_** ligand is shown for comparison; Figure S2. The lump power–voltage curve (a) and current density–voltage dependence (b) for the OLED structure of type **A** with **Nd1** complex; Figure S3. Electroluminescence spectra for the **B** devices based on the spin-coated **Nd2** complex in **dmfl-CBP:TCTA** (7:3, 2000 rpm), **PVK** (2000 rpm), and **TCTA** (2000 rpm) matrices; Figure S4. EQE–radiant power ratio for **B** OLEDs based on **Nd1**-**Nd3** complexes; Figure S5. Visible electroluminescence spectra for the type **B** devices at 5 V (solid line) based on the **Nd2** complex, [Gd(L_2_)phen] complex with a (1:1) host:guest ratio and TCTA as the emission layer, and for similar devices with TCTA, [Gd(L_2_)phen] and the **Nd2** complex, but a BPhen transport layer instead of TPBi, at 9 V. The photoluminescence spectrum of the neat **HL_2_** ligand is shown for comparison; Figure S6. Calculated frontier molecular orbitals with the largest contribution in T_1_ state for the **Nd1** complex; Figure S7. Morphology of the spin-coated films with neodymium complexes; Table S1. Weight loss for the **Nd1**-**Nd3** complexes (DTA/TGA analysis); Table S2. Optimized geometry of the **Nd1** complex with B3PW91.
