# Supplementary material for: OLED Structure Optimization for Pure and Efficient NIR Electroluminescence of Nd3+ Complexes Bearing Fluorinated 1,3-Diketones
Source: Materials (Basel). 2023 Feb 1;16(3):1243. doi: 10.3390/ma16031243 (PMC9919853; doi:10.3390/ma16031243)
Supplement: Supplementary file 1 [file materials-16-01243-s001.zip › materials-2109127-supplementary.pdf]

Supporting information

OLED Structure Optimization for Pure and Efficient NIR Electroluminescence of Nd^{3+} Complexes Bearing Fluorinated 1,3-Diketones

Daria A. Metlina [1], Dmitry O. Goryachii [1], Mikhail T. Metlin [1], Lyudmila V. Mikhalchenko [2], Vladislav M. Korshunov [1] and Ilya V. Taydakov [1,3,*]

[1] P.N. Lebedev Physical Institute of the Russian Academy of Sciences, 53 Leninsky Prospect, 119991 Moscow, Russia
[2] N.D. Zelinsky Institute of Organic Chemistry of the Russian Academy of Sciences, 47 Leninsky Prospect, 119991 Moscow, Russia
[3] G.V. Plekhanov Russian University of Economics, 36 Stremyanny per., 117997 Moscow, Russia
* Correspondence: taidakov@gmail.com

Synthesis of complexes

Tris-(1-(1,3-dimethyl-1H-pyrazol-4-yl)-4,4,4-trifluorobutane-1,3-dionato) (1,10-phenanthroline) neodymium(III) (**Nd1** complex), tris-(1-(1-methy-1H-pyrazol-4-yl)-4,4,5,5,6,6,6-heptafluorohexane -1,3-dionato) (1,10-phenanthroline) neodymium(III) (**Nd2** complex) and tris-(4,4,5,5,6,6,7,7,8,8,9,9,9- tridecafluoro-1-(1-methyl-1H-pyrazol-4-yl)nonane-1,3-dionato) (1,10-phenanthroline) neodymium(III) (**Nd3** complex), see Figure 1, were synthesized as reported previously [29] with minor modifications.

Neodymium oxide Nd_2O_3 (99.99%, 2.102 g, 6.25 mmol) was dissolved in a minimum amount of concentrated HCl (high purity grade). The solution was evaporated to dryness on a water bath and the residue was transferred to a volumetric flask. The volume was brought to 25 mL with deionized water to obtain a 0.5 M stock solution of $NdCl_3$.

The Nd1 complex

The HL1 (0.351 g, 1.5 mmol) and 1,10-phenanthroline (0.09 g, 0.5 mmol) ligands were dissolved in warm ethanol (8 mL); 0.5 M solution of $NdCl_3$ (1 mL) and then 1 M NaOH (1.5 mL) were added dropwise with stirring. The resulting greenish-blue solution was filtered, stored at 55°C for 2 h, and kept for 24 h at room temperature. The solvent was then evaporated to dryness under reduced pressure and the residue was extracted with 30 mL of warm acetone. The solution was filtered and left in an open beaker at room temperature. After evaporation of the solvent, the glassy residue was re-dissolved in a minimum amount of warm MeCN. Crystallization was induced by careful addition of an equal volume of Et_2O while rubbing the walls with a glass rod. The pale greenish powder was separated, washed with hexane, and dried under reduced pressure (50 °C and 0.1 Torr) to a constant weight. The yield was 0.347 g (67%). For $C_{39}H_{32}F_9N_8NdO_6$ (FW 1023.95)

Calcd., %: C, 45.75; H, 3.15; N, 10.94; Nd, 14.09.
Found, %: C, 45.91; H, 3.23; N, 11.09; Nd, 14.24.
IR ν (cm^{-1}): 1657, 1540, 1465, 1345.
NMR assignment: ^1H NMR (CD_2Cl_2), δ (ppm): -0.6 (br. s, 9H, CCH_3), 1.26 (br. s, 2H, 2H-phen), 3.92 (br. s, 9H, N-CH_3), 5.89 (br. s, 2H, 3H/4H-phen), 6.15 (br. s, 2H, 3H/4-phen), 6.52 (br. s, 2H, 6H-phen), 9.46 (br. s, 3H, 5H-pyr), 10.88 (br. s, 3H, CH-acac). $^{19}F\{^1H\}$ NMR (CD_2Cl_2), δ (ppm): -75.68 (s, CF3).
The Nd2 complex
This complex was prepared similarly to complex **Nd1** from HL2 (0.384 g, 1.2 mmol), 1,10-phenanthroline (0.072 g, 0.4 mmol), 0.8 mL of the $NdCl_3$ stock solution, and 1.2 mL of 1 M NaOH. The precipitate formed on standing was separated, extracted with 30 mL of warm acetone, and the pure solution was evaporated in an open beaker at room temperature. The crystalline powder was collected, washed with water, 30% aqueous EtOH, and hexane, and dried under reduced pressure (50◦C and 0.1 Torr) to a constant weight. The yield of the pale greenish-blue powder was 0.397 g (77%).
For $C_{44}H_{26}F_{21}N_8NdO_6$ (FW 1281.91)
Calcd., %: C, 39.35; H, 2.04; N, 8.74; Nd, 11.25.
Found, %: C, 39.48; H, 2.15; N, 8.91; Nd, 11.39.
IR ν (cm^{-1}): 1657, 1540, 1465, 1345.
NMR assignment: ^1H NMR (CD_2Cl_2), δ (ppm): 1.26 (br. s, 2H, 2H-phen), 3.77 (br. s, 9H, N-CH3), 6.02 (br. s, 2H, 3H/4H-phen), 6.12 (d, 3 JH–H = 7.7 Hz, 2H, 3H/4H-phen), 6.62 (d, 3 JH–H = 7.7 Hz, 2H, 6H-phen), 7.87 (br. s, 3H, 3H-pyr), 8.33 (br. s, 3H, 5H-pyr), 11.14 (br. s, 3H, CH-acac). $^{19}F\{^1H\}$ NMR (CD_2Cl_2), δ (ppm): -126.32 (br. s, 2F, 1-CF_2), -116.00 (q, 3 JH–H = 7.1 Hz, 2F, 2-CF_2), -81.05 (t, 3 JH–H = 7.1 Hz, 3F, CF_3).
The Nd3 complex
This complex was prepared similarly to complex **Nd1** from HL3 (0.423 g, 0.9 mmol), 1,10-phenanthroline (0.054 g, 0.3 mmol), 0.6 mL of a $NdCl_3$ stock solution, and 0.9 mL of 1 M NaOH. A gel formed on standing. The reaction mixture was diluted with 50 mL of CH_2Cl_2, filtered, and the precipitate was washed with 30 mL of hot EtOH. The solvent was evaporated to dryness under reduced pressure. The solid residue was extracted with 15 mL of acetone, filtered through a 0.45 μm Teflon syringe filter, and left in an open beaker at room temperature. The crystalline powder was collected, washed with water, 30% aqueous EtOH, and hexane, and dried under reduced pressure (50◦C and 0.1 Torr) to a constant weight. The yield of the pale greenish-blue powder was 0.355 g (68%).
For $C_{51}H_{26}F_{39}N_8NdO_6$ (FW 1731.98)
Calcd., %: C, 35.37; H, 1.51; N, 6.47; Nd, 8.33.
Found, %: C, 35.59; H, 1.63; N, 6.47; Nd, 8.60.
IR ν (cm^{-1}): 1657, 1540, 1465, 1345.
NMR assignment: ^1H NMR (CD_2Cl_2), δ (ppm): 1.25 (br. s, 2H, 2H-phen), 3.74 (br. s, 9H, N-CH_3), 5.99 (br. s, 2H, 3H/4H-phen), 6.07 (br. s, 2H, 3H/4H-phen), 6.60 (br. s, 2H, 6H-phen), 7.77 (br. s, 3H, 3H-pyr), 8.52 (br. s, 3H, 5H-pyr), 11.14 (br. s, 3H, CH-acac). $^{19}F\{^1H\}$ NMR (CD_2Cl_2), δ (ppm): -126.64 (br. s, 2F, 1-CF_2), -123.24 (br. s, 2F, 2-CF_2), -121.84 (br. s, 4F, 3,4-CF_2), -115.04 (br. s, 2F, 5-CF_2), -81.35 (br. s, 3F, CF_3).

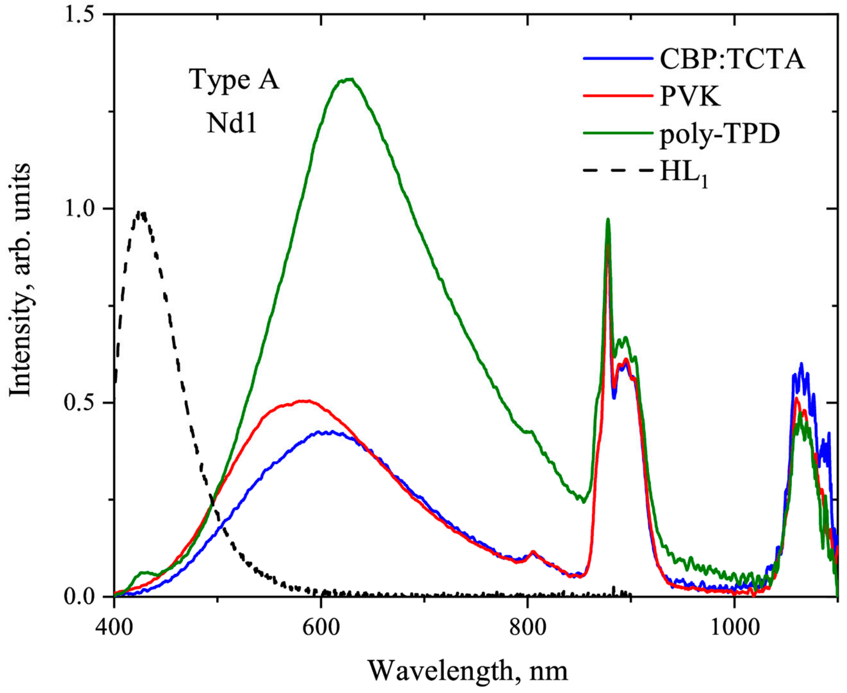

Figure S1. Electroluminescence spectra for the **A** type devices based on the **Nd1** complex with **dmfl** **CBP:TCTA**(7:3, 2000 rpm) (1), **PVK**(2000 rpm)(2) and **polyTPD** (2000 rpm) (3). The photoluminescence spectrum of the neat **HL**$_1$ ligand is shown for comparison.

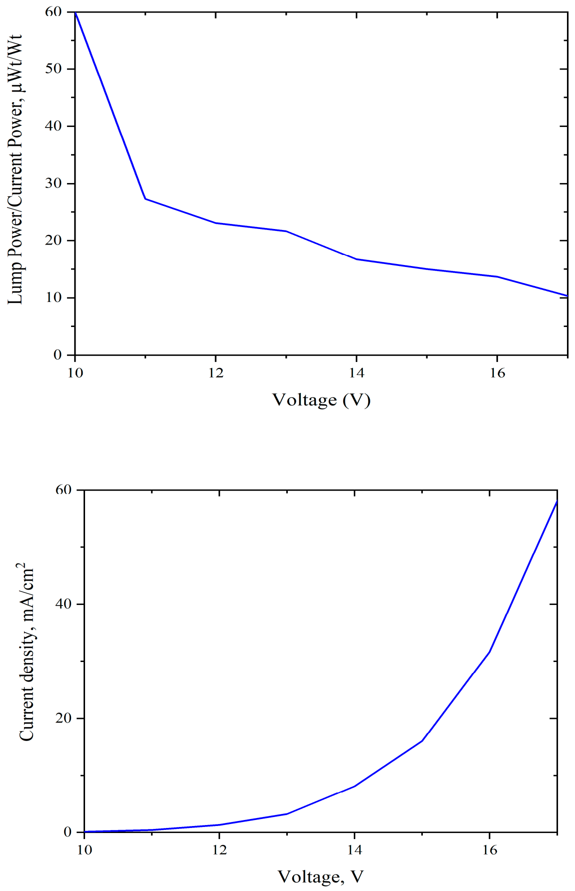

a b

Figure S2. The lump power-voltage curve (a) and current density – voltage dependence (b) for the OLED structure of type **A** with **Nd1** complex**.**

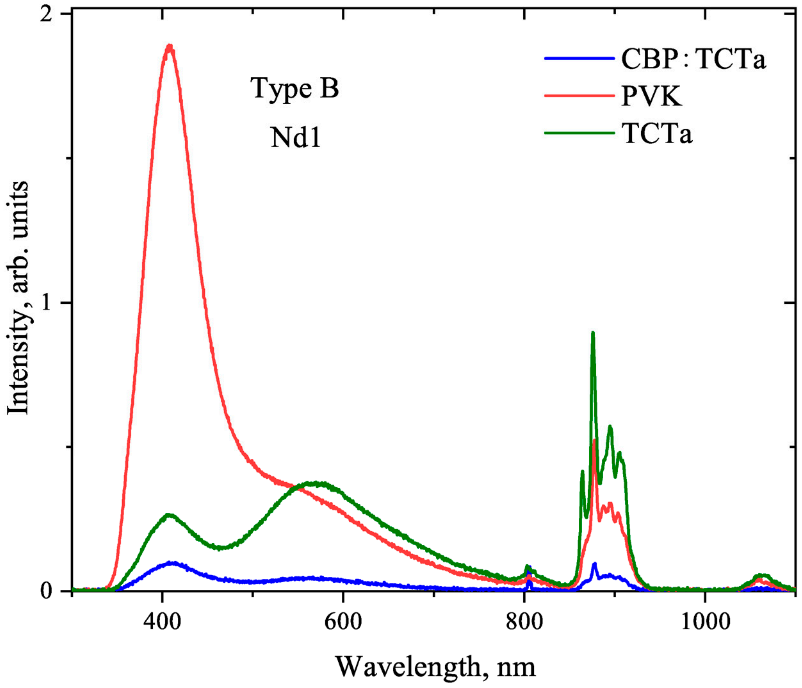

Figure S3. Electroluminescence spectra for the **B** devices based on the spin coated **Nd2** complex in **dmfl-CBP:TCTA** (7:3, 2000 rpm), **PVK**(2000 rpm) and **TCTA** (2000 rpm) matrices.

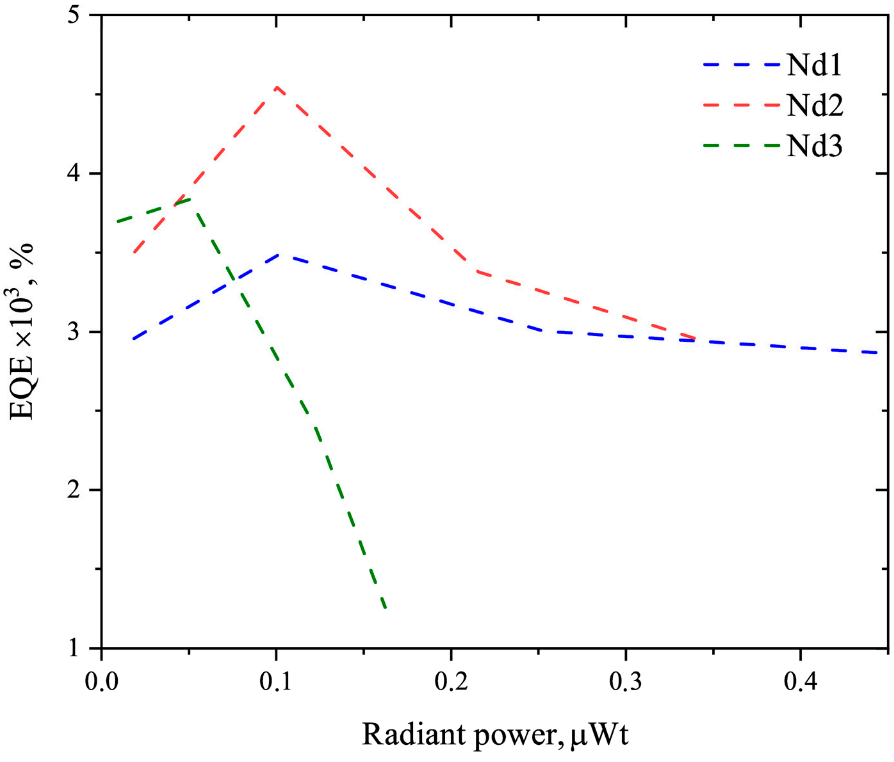

Figure S4. EQE-Radiant power ratio for **B** OLEDs based on **Nd1 - Nd3** complexes.

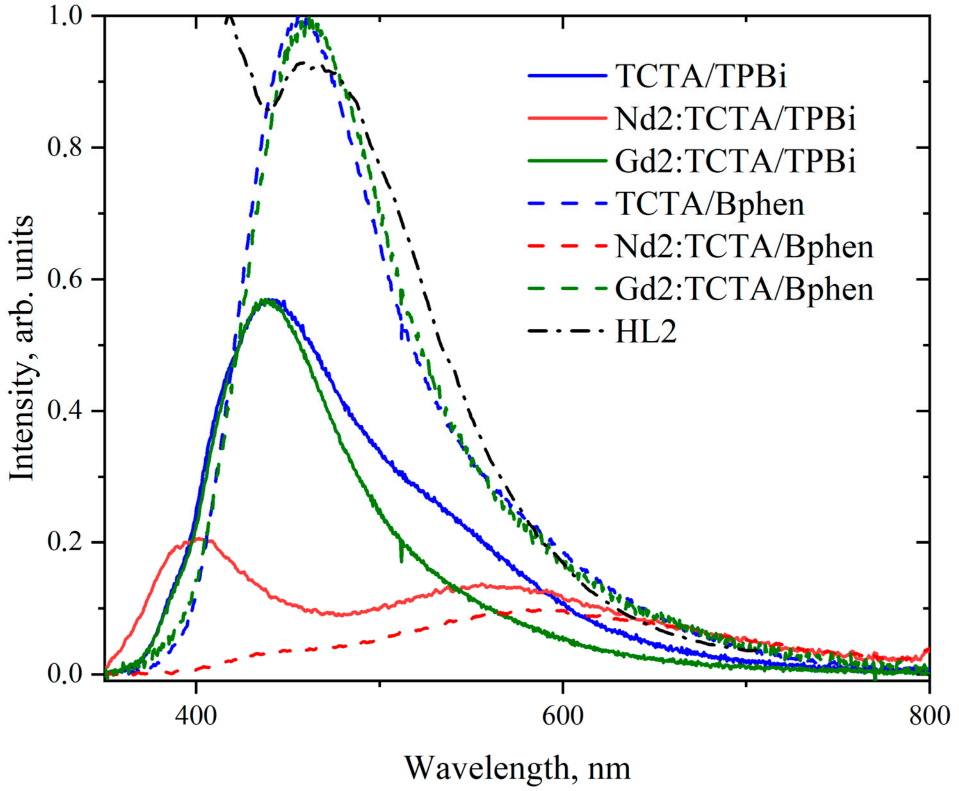

Figure S5. Visible electroluminescence spectra for the type **B** devices at 5V (solid line) based on the **Nd2** complex, Gd(HL2)phen complex with a (1:1) host:guest ratio and TCTA as the emission layer, and for similar devices with TCTA, Gd(HL2)phen and the **Nd2** complex, but a BPhen transport layer instead of TPBi, at 9V. The photoluminescence spectrum of the neat HL2 ligand is shown for comparison.

Table S1. Weight loss for the Nd1-Nd3 complexes (DTA/TGA analysys).

Sample	Initial themperature, ◦C	Weight loss, %	Calculated weigth loss, %
**Nd1**	225	67	68
**Nd2**	250	75	75
**Nd3**	190	77	82

Table S2. Optimized geometry of the **Nd1** complex with B3PW91

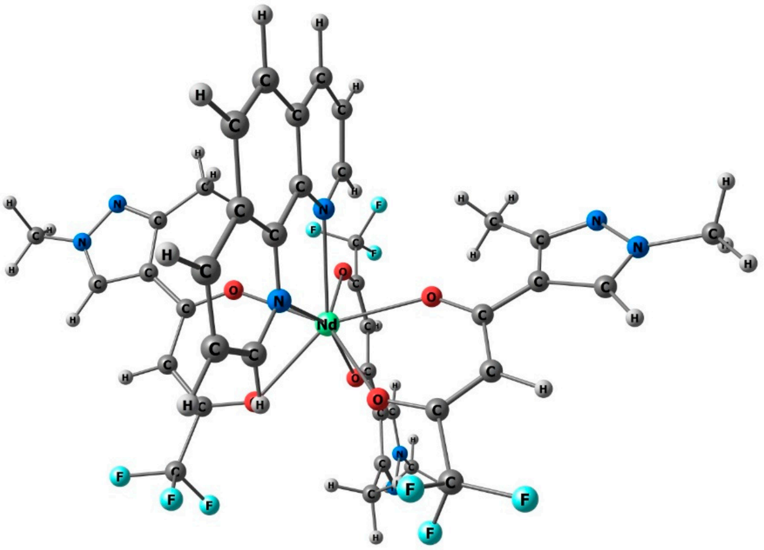

Nd	10.820176000	7.251296000	7.999882000
F	7.509832000	3.275097000	7.757047000
F	6.547184000	3.591631000	9.670807000
F	8.622534000	2.980760000	9.591451000
F	9.710805000	10.607305000	3.313049000
F	11.208761000	11.185872000	4.766009000
F	9.109294000	11.200504000	5.305224000
O	11.489945000	6.785146000	10.343796000
O	11.866596000	9.216892000	9.007111000
O	9.352214000	5.345101000	8.489399000
O	8.683881000	8.041922000	8.829458000
O	10.468270000	9.022380000	6.360523000
O	10.412278000	6.293062000	5.756171000
N	12.809346000	6.060741000	14.603926000
N	4.755225000	9.491007000	10.492469000
N	5.564143000	10.527682000	10.181859000
N	9.442261000	4.648905000	1.664565000
N	9.704909000	3.651245000	2.535132000
N	12.023316000	5.051248000	14.175186000
C	12.020907000	7.381599000	11.320708000
C	12.459763000	8.736378000	11.267204000
H	12.882033000	9.187069000	12.152232000

C	12.347208000	9.517780000	10.132256000
C	12.195136000	6.647978000	12.576790000
C	12.934963000	7.033648000	13.689413000
H	13.539002000	7.908004000	13.877788000
C	11.644455000	5.384878000	12.948169000
C	10.741199000	4.487194000	12.178202000
H	11.234576000	4.114026000	11.278826000
H	9.848107000	5.020776000	11.846598000
C	13.402059000	5.989902000	15.919962000
H	13.973699000	6.900265000	16.100365000
H	14.064655000	5.124418000	15.986328000
H	12.618368000	5.897252000	16.674229000
C	12.874497000	10.963786000	10.196481000
C	7.585643000	7.599832000	9.264573000
C	7.286775000	6.204991000	9.332222000
H	6.320724000	5.891491000	9.697763000
C	8.175604000	5.225793000	8.936478000
C	6.583612000	8.562440000	9.707601000
C	5.318759000	8.305061000	10.227424000
H	4.798541000	7.379470000	10.421045000
C	6.677881000	9.988919000	9.704763000
C	7.799881000	10.859774000	9.260046000
H	7.513682000	11.906176000	9.376668000
H	8.060076000	10.667558000	8.217403000
H	8.702423000	10.666029000	9.843812000
C	3.446280000	9.755346000	11.044544000
H	2.940342000	8.807312000	11.227391000
H	2.860648000	10.351606000	10.342063000
H	3.542839000	10.304156000	11.983449000
C	7.699778000	3.762981000	8.998338000
C	10.175550000	9.081577000	5.134704000
C	9.985077000	8.051612000	4.233740000
H	9.732692000	8.309658000	3.216715000
C	10.116183000	6.677253000	4.592388000
C	9.896695000	5.662446000	3.560965000
C	9.543517000	5.860141000	2.230045000
H	9.358327000	6.760602000	1.664763000
C	9.981209000	4.243035000	3.689644000
C	9.088349000	4.326372000	0.301091000
H	8.923700000	5.251942000	-0.250536000
H	8.176572000	3.725904000	0.284981000
H	9.895728000	3.761158000	-0.168601000
C	10.321258000	3.422543000	4.883995000

H	10.239785000	2.364188000	4.630256000
H	9.657009000	3.652043000	5.718939000
H	11.338525000	3.634085000	5.222617000
C	10.042375000	10.526107000	4.617624000
N	12.493705000	5.116763000	7.853043000
N	13.299001000	7.559720000	6.938120000
C	12.096904000	3.932937000	8.292134000
H	11.084144000	3.889328000	8.680118000
C	12.909303000	2.789074000	8.251312000
H	12.526590000	1.847765000	8.627760000
C	14.175274000	2.893207000	7.727529000
H	14.833279000	2.030749000	7.677894000
C	14.625283000	4.136795000	7.243118000
C	15.926315000	4.318066000	6.677100000
H	16.590885000	3.461580000	6.621969000
C	16.324052000	5.531932000	6.217701000
H	17.312496000	5.663558000	5.788797000
C	15.453309000	6.664158000	6.287868000
C	15.825863000	7.941673000	5.825607000
H	16.811301000	8.086381000	5.392904000
C	14.939460000	8.986473000	5.931463000
H	15.193026000	9.983112000	5.590441000
C	13.678777000	8.749776000	6.501348000
H	12.955216000	9.550399000	6.612412000
C	14.160046000	6.523542000	6.842293000
C	13.735319000	5.233059000	7.329837000
F	13.840084000	11.153234000	9.274586000
F	13.404468000	11.295708000	11.391007000
F	11.896647000	11.844653000	9.938655000
H	10.446747000	3.643207000	12.803924000

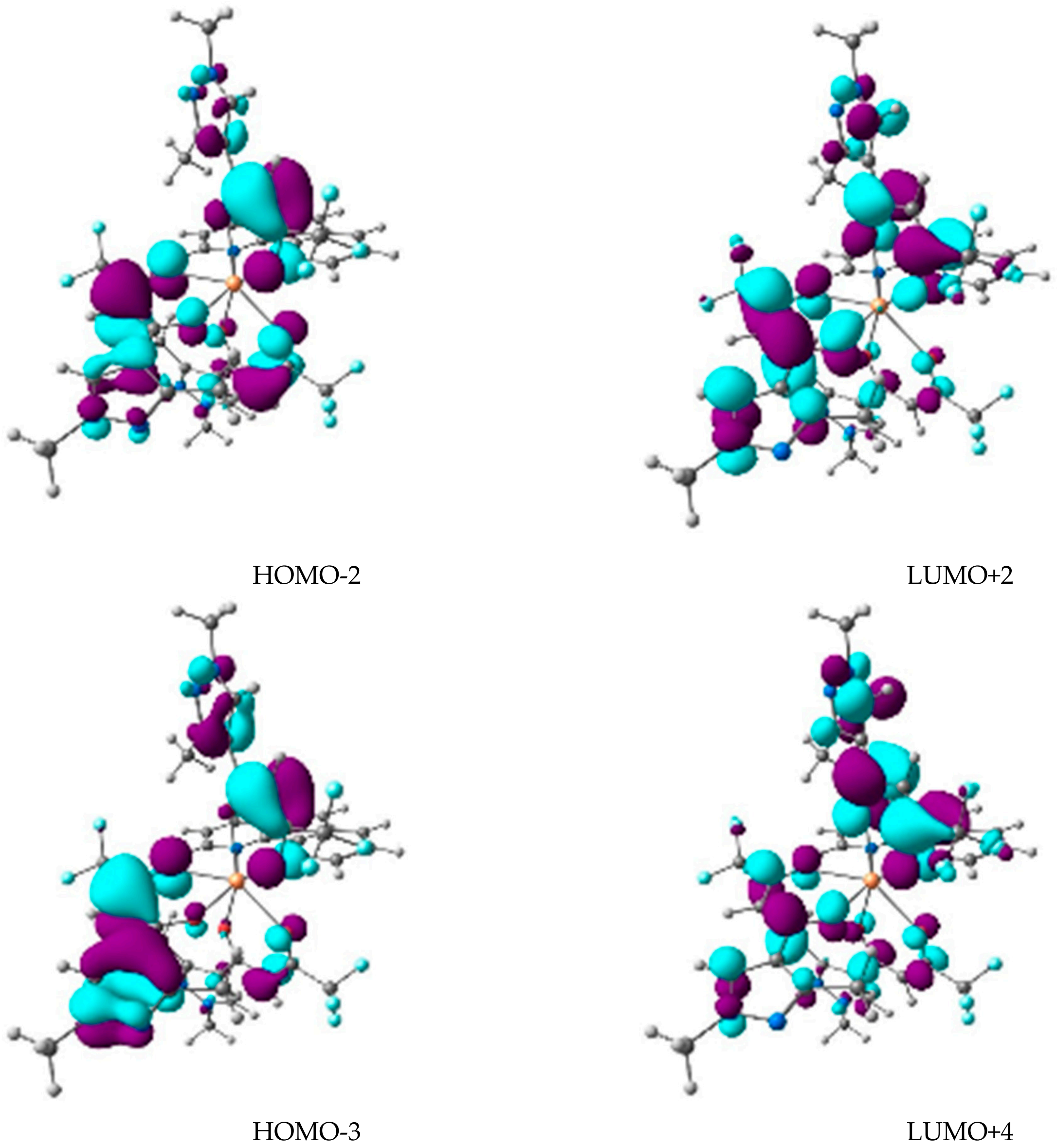

Figure S6. Calculated frontier molecular orbitals with the largest contribution in T_1 state for the **Nd1** complex.

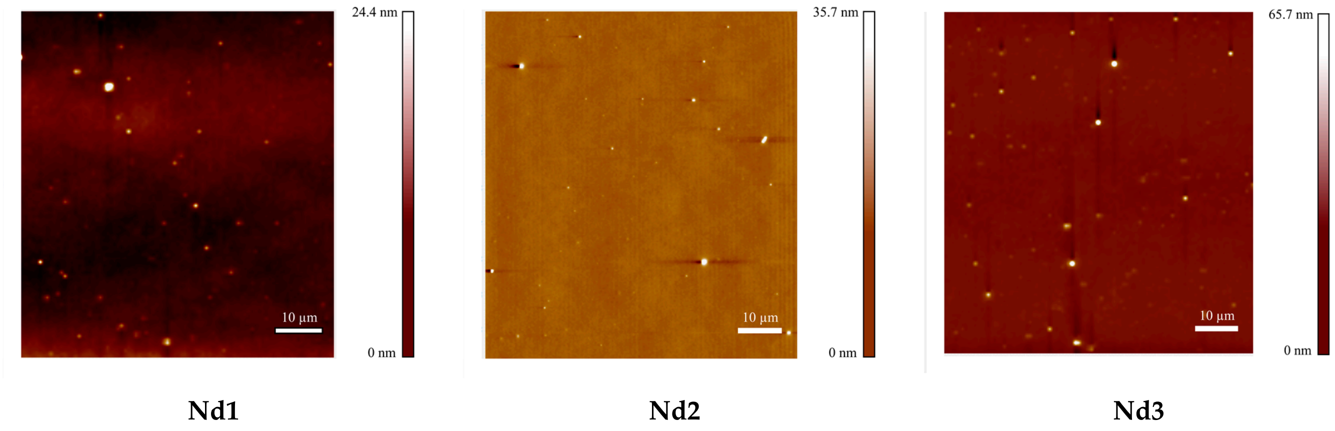

Figure S7. Morphology of the spin-coated films with neodymium complexes.

References

1. Metlina DA, Metlin MT, Ambrozevich SA, et al (2020) Bright NIR-luminescent Nd3+ complexes with pyrazole-substituted 1, 3-diketones demonstrated an unusual spectral lines branching ratios. Dyes and Pigments 181:108558
